# Physical Education

**Published:** 1859-03

**Authors:** 


### PHYSICAL EDUCATION.

Dr. Cox is reported to have said in a college address: "I am glad that Luther had a good digestion as well as a great soul, for the reformation would have been delayed had he been a dyspeptic." The rev. doctor has been a martyr himself to throat ail, arising from a dyspeptic stomach; and it has been reported to us that his wife is the only person able to keep him well, by always accompanying him and treading on his big toe under the table to remind him that he had eaten enough, and instanter the plate is obediently pushed back.

